# Initial characterization of immune microenvironment in pheochromocytoma and paraganglioma

**DOI:** 10.3389/fgene.2022.1022131

**Published:** 2022-12-07

**Authors:** Bo Jin, Wencong Han, Jingjing Guo, Jie Tian, Shiming He, Yanqing Gong, Jingcheng Zhou, Qun He, Qi Shen, Zheng Zhang

**Affiliations:** ^1^ Department of Clinical Laboratory, Peking University First Hospital, Beijing, China; ^2^ Department of Urology, Peking University First Hospital, Peking University, Beijing, China; ^3^ Institute of Urology, Peking University, Beijing, China; ^4^ National Urological Cancer Center, Beijing, China

**Keywords:** pheochromocytoma, immune microenvironment, PD-L1, immunotherapy, microsatellite instability

## Abstract

Due to fewer adverse events, faster onset of action, and longer durable responses compared to chemotherapy, immunotherapy has been widely used to treat advanced solid tumors. Moreover, immunotherapy can improve the autoimmune status, thus allowing patients to benefit from the treatment in the long term. The immune microenvironment status is closely associated with the response to chemotherapies. Here, we analyzed the characteristics of the immune microenvironment in pheochromocytoma and paraganglioma (PPGL). Immunohistochemistry showed that PD-L1 is sparely expressed in PPGL with low positive rates and low expression levels, an expression pattern, that is, not correlated with tumor malignancy. Moreover, the level of intratumoral CD4^+^ and CD8^+^ lymphocyte infiltration in PPGL is low, suggesting that the immune microenvironment in PPGL may be in “immune desertification” or “immune rejection” states in which CD4^+^ and CD8^+^ lymphocyte infiltration is prevented, rendering immunotherapy less effective. In sum, our results indicate that PPGL is a microsatellite-stable tumor with low tumor mutational burden (TMB) levels, weak neoantigen production, and poor tumor antigenicity, hinting at a poor response of PPGL to chemotherapies.

## Introduction

In 2017, as per the revised WHO pathological classification, pheochromocytoma and paraganglioma (PPGL) have been classified as malignant tumors. It is recommended to drop the use of “benign or malignant PPGL” and instead adopt the “primary and metastatic malignancy” classification (Mete et al., 2022). Currently, surgical resection is a standard treatment for local PPGL, but for metastatic PPGL, the standard treatment is still not available yet (Plouin et al., 2016). Targeted therapy, combinatory chemotherapy, and 131I-MIBG have been used to treat metastatic PPGL, resulting in tumor shrinkage, disease stabilization, and symptom amelioration (Granberg et al., 2021). However, the responses of tumors to these treatments are highly variable, and the efficacy of therapies is greatly affected by the underlying status of patients. Therefore, new systemic therapies that can alleviate or cure the metastasis of PPGL are urgently needed.

Because of fewer adverse events, faster onset of action, and longer durable responses than chemotherapy, immunotherapy has been widely used to treat advanced solid tumors (Tan et al., 2020). Autoimmune disorders are closely associated with cancer development (Giat et al., 2017). Cancer patients are predisposed to develop autoimmune disorders, and *vice versa* (Giat et al., 2017). Therefore, improving autoimmune status can enhance the efficacy of immunotherapy. However, the characteristics of the tumor immune microenvironment in PPGL have been understudied, and the efficacy of immunotherapy to treat PPGL is unclear (Batchu et al., 2022). Cancer cells have the ability to activate different immune checkpoint pathways that are involved in immunosuppression. Hence the development of monoclonal antibodies to block the activation of immune checkpoints is a revolutionary milestone in the field of immuno-oncology. Among the immune checkpoint inhibitors (ICIs) developed so far, pembrolizumab and nivolumab that target PD-1/PD-L1 have shown promising therapeutic potentials with high objective response rates (ORR) in a variety of cancers including melanoma and non-small cell lung carcinoma, urothelial bladder cancer, and triple-negative breast cancer (Darvin et al., 2018). Largely, the effect of ICIs relies on tumor antigenicity, PD-L1 expression levels, and tumor lymphatic infiltration status of the immune microenvironment of tumor cells.

Neoantigens are tumor-specific antigens generated by somatic mutations in tumor cells (Heemskerk et al., 2013). Because of their exclusiveness to tumor cells and high immunogenicity, neoantigens serve as biomarkers by which immune cells recognize tumor cells (Lee et al., 2018). Tumor antigenicity determines the efficacy of immune therapy and has become a new direction in immunotherapy research (Ott et al., 2017). Empirical evidence suggests that tumor mutational burden (TMB) is the main drive for neoantigen production (Schumacher and Schreiber, 2015). TMB is defined as the number of somatic, coding, indel mutations, and base substitutions per megabase (Mb) of the genome examined. Patients with high tumor mutational burden (TMB-H) have numerous genetic mutations that allow tumor cells to produce high numbers of neoantigens (Jardim et al., 2021). TMB-H is primarily caused by tumor microsatellite instability-high (MSI-H) which mainly results from DNA deficient mismatch repair (dMMR) (Shao et al., 2020).

Programmed death-ligand 1, CD274 (PD-L1), is a ligand of programmed cell death protein 1 (PD1). PD-L1 acts as an inhibitor of human T-cell responses (Dong et al., 1999), protecting tissues from autoimmune attacks. Under normal physiological conditions, *PD-L1* mRNAs are expressed in various tissues including the placenta, vascular endothelium, pancreatic islet cells, muscle, hepatocytes, epithelium, and mesenchymal stem cells, as well as immune cells such as B cells, T cells, dendritic cells, macrophages, and mast cells (Sharpe et al., 2007). Under normal physiological conditions, the PD-L1 proteins are rarely found on cell surface. In stark contrast, PD-L1 proteins are often highly distributed on the surface of tumor cells or in the tumor microenvironments of various cancers, such as gastric cancer (Saito et al., 2018), ovarian cancer (Webb et al., 2016), and lymphoma (Xie et al., 2019). PD-L1 expression levels significantly correlate with the efficacy of immune checkpoint inhibitors and are a reliable biomarker of immunotherapy efficacy (Bagchi et al., 2021).

Activation of T lymphocytes in tumor tissue is a prerequisite for strong immunity (Okla et al., 2021). Therefore, tumor lymphatic infiltration status (TLIs) is closely associated with the efficacy of immune checkpoint inhibitors and the prognosis of cancers (Liu et al., 2021).

Here, we probed the relationship between PD-L1 expression, TIL status, and TMB/MSI-H/MMR in different PPGL cases and preliminarily described the basic characteristics of the immune microenvironment in PPGL. Our results suggest that PPGL may be in “immune desertification” or “immune rejection” states in which lymphocyte infiltration is blocked, which could greatly impair the efficacy of immunotherapy in PPGL treatment.

## Materials and methods

### Tumor tissues and clinical data

A total of 72 patients with PPGL who undertook surgical treatment in the Department of Urology, Peking University First Hospital from 2007 to 2019 were enrolled in this study. Among them, 47 had non-metastatic PPGL, six had recurrent PPGL, and 19 had metastatic PPGL. The baseline demographic characteristics were shown in Table 1 **.** The diagnosis of PPGL was mainly based on clinical manifestations and a family history of related diseases. Metastatic PPGL was defined as the presence of metastases in the tissues apart from the adrenal medulla revealed by computed tomography (CT). Paraffin-embedded tumor specimens from the patients were obtained from the tumor tissue bank of the Department of Pathology and Urology, Peking University First Hospital.

**TABLE 1:** Baseline demographic characteristics and Pheochromocytoma of the Adrenal Gland Scoring Scale (PASS).

Characteristics	Number (%)
Gender	
Male	40 (55.6%)
Female	32 (42.4%)
Age (years)	46 (33–58)
Tumor size (cm)	5.5 (3.9–8.0)
Tumor Location	
Adrenal glands	44 (61.1%)
Ectopic	28 (38.9%)
Tumor Specimen Source	
Recurrent Group	6 (7.4%)
Primary foci in the metastatic group	19 (23.5%)
Metastatic foci in the metastatic group	9 (11.1%)
Non-metastatic and recurrent group	47 (58.0%)
Features	PASS Scores
Large nests or diffuse growth (>10% of tumor volume)	2
Central (middle of large nests) or confluent tumor necrosis (no degenerative change)	2
High cellularity	2
Cellular monotony	2
Tumor cell spindling (even if focal)	2
Mitotic figures >3/10 HPF	2
Atypical mitotic figures	2
Adipose tissue invasion	2
Vascular invasion	1
Capsular invasion	1
Profound nuclear pleomorphism	1
Nuclear hyperchromasia	1
Total	20

### Patient classification

The PASS (Pheochromocytoma of the Adrenal Gland Scaled Score, PASS) scoring system was used to determine the tumor malignancy in 29 cases of non-metastatic PPGL (Table 1). Patients were divided into low metastatic potential (PASS-L) and high metastatic potential (PASS-H) groups based on a cut-off score of 4. The clinicopathological data of the 29 patients with PPGL were analyzed with 12 independent variables: large nests or diffuse growth (>10% of tumor volume), central or confluent necrosis, high cellularity, cellular monotony, tumor cell spindling, mitosis figures (>3/10 HPF), atypical mitotic figures, adipose tissue invasion, vascular invasion, capsular invasion, profound nuclear pleomorphism, and nuclear hyperchromasia.

### Characterization of immune microenvironment in PPGL

The paraffin-embedded specimens of PPGL were serially sectioned at 4 μm. Then the sections were treated with freshly prepared dimethylbenzene for 10 min at room temperature, followed by treatment with 100% ethanol for 2 min, 95% ethanol for 2 min, and 75% ethanol for 2 min. After a brief wash in double-stilled water, the sections were placed in 1 × PBS buffer (pH 7.4). Next, the sections were treated with EDTA antigen retrieval solution (Origene, Beijing, China) and in a pressure cooker on high pressure (approximately120°C) for 15 min. Then the slides were cooled down in a pressure cooker for 10 min before releasing pressure, followed by the incubation on host double distilled water for 2 min. Subsequently, the sections were placed in room temperature 1 × PBS buffer for 2 min and were treated with 3% H_2_O_2_ for 10 min at room temperature. The sections were then stained overnight at 4°C with hematoxylin-eosin (HE) and DAKO 28–8 (anti-PD-L1), EPR6855 (anti-CD4), CAL67 (anti-CD8), anti-MLH1, anti-MSH2, anti-MLH6, and anti-PMS2, respectively. After two washes with 1 × PBS, the sections were treated with goat-anti-rabbits IgGs at 37°C for 30 min. Following two washes with 1 × PBS, the immune reactions were visualized by DAB buffer (Origene, Beijing, China). DAKO 28–8 was obtained from Dako North America (United States), and EPR6855 and CAL67 were purchased from Abcam (United States). Other primary antibodies were obtained from Origene (Beijing, China). All primary antibodies were derived from rabbits and diluted 500 folds. Immunohistochemical staining of PD-L1, CD4, and CD8 was performed on tumor tissues from 14 patients with PASS-L, 15 patients with PASS-H, 6 patients with recurrent PPGL, and 19 patients with metastatic PPGL. In the 9 cases of metastatic PPGL, both primary and metastatic tumor specimens were collected. The same experiment without application of the primary antibodies was used as negative controls. The expression of PD-L1 and intratumoral and stromal CD4^+^ and CD8^+^ lymphocyte infiltration were analyzed in the 63 cases of PPGL with different degrees of malignancy. To examine PD-L1 expression, samples containing more than 100 living tumor cells were chosen. The combined positive score (CPS) calculates the ratio of tumor cells, lymphocytes, and macrophages that are expressing PD-L1 relative to the total number of viable tumor cells. CPS scores of ≥1 were considered positive for PD-L1 expression. To assess the infiltration of CD4^+^ and CD8^+^ lymphocytes, the percentage of CD4^+^ or CD8^+^ lymphocytes was calculated against the total amount of lymphocytes. The levels of lymphocytic infiltration of CD4 and CD8 were determined as follows: 1) no positive cells or few dispersed positive cells; 2) infiltration of less than 25% of the stromal area or sparsely dispersed positive cells across the entire core area; 3) infiltration of 25% to 49% of the stromal area; 4) infiltration of 50% or greater of the stromal area. The a-c infiltration levels were considered low, and the d level was considered high.

To detect MSI and dMMR, fluorescent PCR-capillary electrophoresis was performed using an MSI detection kit (Microread, Beijing, China) as per the manufacturer’s instructions. Briefly, the volume of PCR reactions was 20 μl comprising 10 μl of reaction buffer, 0.5 μl of DNA polymerases, 4 μl of MSI primers, 1 μl of genomic DNA, and 4.5 μl of nucleotide-acid-free water. The PCR reaction was run as follows: UDG digestion for 10 min, followed by pre-denaturation at 95°C for 5 min, 30 cycles of denaturation at 94°C for 30 s, annealing at 60°C for 1 min, and elongation at 70°C for 1 min, followed by post-elongation at 60°C for 30 min. To do capillary electrophoresis, 10 μl of sample comprising 8.7 μl of deionized-formamide, 0.3 μl of internal lane standard ROX 500, and 1 μl of PCR products was applied to the Applied Biosystems 3500 Genetic Analyzers (ThermoFisher Scientific, United States). The blood samples and frozen specimens of fresh tumor tissues were collected from 18 patients with PPGL undergoing surgical treatment in the Department of Urology, Peking University First Hospital from 2018 to 2019. Six regions of mononucleotide repeats including NR-21, BAT-26, NR-27, BAT-25, NR-24, and MONO-27 were used for MSI determination and two pentanucleotide markers (penta C and penta D) were used as controls.

To detect TMB, whole-exome sequencing was performed with 16 patients. TMB was defined as the number of somatic mutations per megabase including SNV and InDel that were filtered according to the following rules: 1) depth greater than 50×; 2) allele frequency greater than 0.05 for the WES panel; 3) not in dbSNP; 4) not in esp6000vis2_all and 1000G_All databases or not recorded by either of the two databases; 5) popular frequency lower than 0.01; 6) inclusion of synonymous, nonsynonymous, frameshift and nonframeshift mutations; 6) exclusion of mutations in the blacklist. To avoid counting mutations present in neighbor 3-bp repeatedly, we only counted once for this event. The estimated TMB for a sample detected by WES was defined as being equal to the total mutation frequency/38 Mb because 38 Mb is commonly considered the typical length of a human exon. Mutations with allelic fractions less than 0.05 or coverage ≤50× were excluded. The percentile of TMB was calculated by the TMB value in a pool of TMB values of around 1000 patients. If the percentile was lower than 25%, a “High” value would be assigned. The TMB value was calculated using the Acornmed TMB software (version 1.0).

## Statistics

Statistical analysis was performed with SPSS 20.0 software. The normally distributed measurement data were presented as X ± s, and the data between the two groups were compared by the independent samples *t*-test. Nonnormally distributed measurement data were presented as median (upper and lower quartiles) and were compared by nonparametric tests. Enumeration data were compared by X^2^ test or exact test. *p* < 0.05 (two-sided test) was considered statistically significant.

## Results

### PASS scores of pheochromocytoma and paraganglioma

The mean age of the 29 patients with PPGL was 49.62 ± 13.35 years with 13 males and 16 females, and the mean tumor size was 6.18 ± 3.41 cm. These 29 cases of PPGL were divided into 14 cases of the PASS-L group and 15 cases of the PASS-H group according to a cut-off of 4. The pathological characteristics of PPGL in the PASS score groups were shown in Table 2.

**TABLE 2:** Pathological characteristics of pheochromocytoma and paraganglioma in PASS score group.

Feature	total, *n*	PASS	
		<4	≥4
Large nests or diffuse growth (>10% of tumor volume)	14	4	10
Central (middle of large nests) or confluent tumor necrosis (no degenerative change)	3	1	3
High cellularity	4	0	4
Cellular monotony	7	2	5
Tumor cell spindling (even if focal)	4	0	4
Mitotic figures >3/10 HPF	3	1	2
Atypical mitotic figures	0	0	0
Extension into adipose tissue	2	0	2
Vascular invasion	4	0	4
Capsular invasion	13	5	8
Profound nuclear pleomorphism	14	5	9
Nuclear hyperchromasia	7	1	6
Total	29	14	15

### PD-L1 expression

**FIGURE 1:**
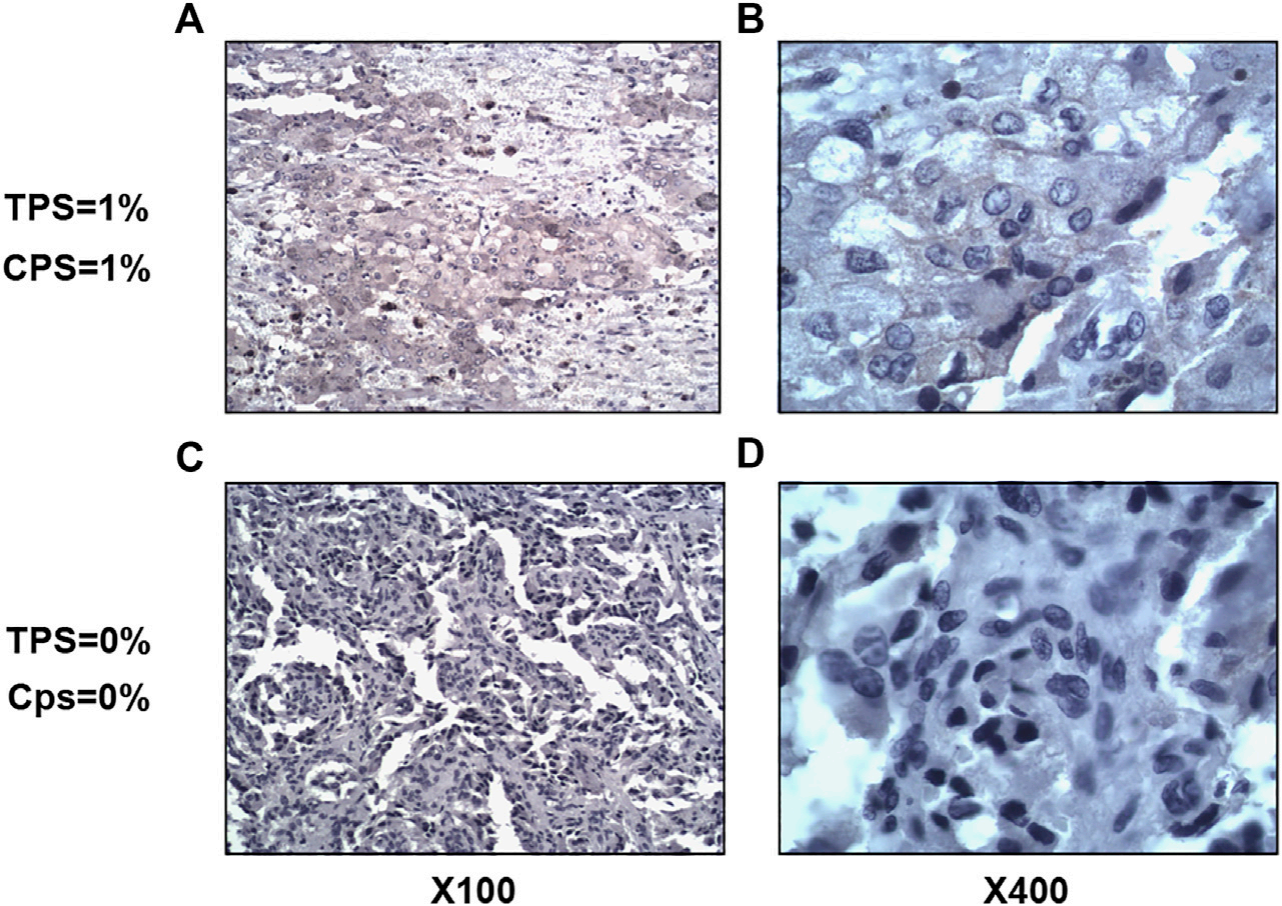
Immunohistochemical staining of PD-L. Left images × 100, right images × 400. **(A,B)**, TPS = 1% and CPS = 1%; **(C,D)**, TPS = 0% and CPS = 0%.

In total, we collected 63 tumor tissue specimens from 54 patients with PPGL, nine of whom contain both primary and metastatic foci. We performed immunohistochemical staining of PD-L1 on these tumor tissue specimens from 14 PASS-L cases, 15 PASS-H cases, six recurrent cases, and 19 metastatic cases. We defined PD-L1 expression as positive in tumor cells by TPS values ≥ 1%. Based on this, the overall positive rate of PD-L1 expression in PPGL was 7.9% (5/63) including 2 cases in the PASS-L group, 2 cases in the recurrent group, and 1 case in the primary foci group (Figure 1). We did not find a significant correlation between the degree of malignancy and PD-L1 expression. The TPS and CPS values of patients with positive PD-L1 expression were 1%.

### Tumor mutational burden levels

We determined the TMB levels in 16 patients including 10 males and six females aged from 15 to 68 years old (Table 3). The pathological feature of each patient and the mutations were presented in Table 3. The TMB levels ranged from 1.8264 to 14.3068. In general, the TMB levels in these patients were low with 11 cases being less than 5 and merely two cases above 10.

**TABLE 3:** Tumor mutational burden levels of 16 patients.

Sample ID (T)	Gender	Age	Symptoms	Location	Size/cm	Germline mutations	Somatic mutations	IHC	TMB
6000000021	Male	68	Palpitation, hyperhidrosis	Left Adrenal Gland	3.2 × 3.2×2.3		HRAS:NM_001130442:exon3:c.182A>G:p.Q61R,HRAS:NM_005343:exon3:c.182A>G:p.Q61R,HRAS:NM_	Vim (+), AE1/AE3 (-), CgA(+), S-100 (+), inhibin (-), CD34 (+), D2-40 (+), Ki67<5%	5.1748
6000000031	Male	50	Hypertension	Left Adrenal Gland	5.5 × 4×4	MAX:NM_145112:exon3:c.C196T:p.R66X,MAX:NM_002382:exon4:c.C223T:p.R75X,MAX:NM_145113:exon4:c.C223T:p.R75X	INSR:NM_001079817:exon13:c.2734C>T:p.R912X,INSR:NM_000208:exon14:c.2770C>T:p.R924X	Vim (+),AE1/AE3 (-),CgA(+),S-100 (+),inhibin (-),CD34 (+),D2-40 (+),Ki67 ˜ 5%	6.3924
6000000061	Male	44	Hypertension	Right Retroperitoneal	6 × 3.2×1.2		TP53:NM_001126115:exon4:c.473G>T:p.R158L,TP53:NM_001126116:exon4:c.473G>T:p.R158L,TP53:NM_	CgA(+++),Syn (+++),NSE(++),S-100 (+),Melan-A (-),Vim (++),AE1/AE3 (+),Ki67 ˜ 1%	4.2616
6000000071	Male	34	Hypertension	Left Adrenal Gland	2.5 × 2.5×2.2			CgA(+),Syn (+),NSE(+),CD34 (+),Ki67<1%	5.1748
6000000081	Male	52	Palpitation, hypertension	Left Adrenal Gland	7 × 5×5			Vim (+),AE1/AE3 (-),CgA(+),S-100 (+),inhibin (-),CD34 (+),D2-40 (+),Ki67<5%	4.8704
6000000101	Male	41	Asymptomatic	Right Adrenal Gland	3.5 × 3.5×3			CgA(+++),Syn (+++),NSE(++),inhibin (-),Melan-A (-),Vim (+),AE1/AE3 (-),Ki67<1%	2.1308
6000000121	Female	15	Palpitation	Right Adrenal Gland	6.3 × 4.5×3.5	VHL:NM_000551:exon2:c.364G>A:p.A122T		CgA(+),NSE(+),Syn (+),CD56 (+),inhibin (-),Melan-A (-),CD34 (+),Ki67 ˜ 5%	1.8264
6000000131	Male	43	Palpitation, hypertension	Bladder	3.8 × 2×2		PIK3C2G:NM_001288772:exon9:c.1339_1343del:p.K447fs,PIK3C2G:NM_001288774:exon9:c.673_	CgA(+++),Syn (++),CD56 (+++),AE1/AE3 (-),CK7(-),SMA (-),CD34 (+++),Ki67<1%	1.8264
6000000141	Male	64	Palpitation, headache, hypertension	Left Adrenal Gland	9 × 8.5×5			CgA(++),Syn (++),inhibin (-),Melan-A (-),Vim (++),AE1/AE3 (+/-),CD34 (+++),Ki67 ˜ 1%	4.5660
6000000001	Male	45	Palpitation, hypertension	Left Adrenal Gland	8 × 7.5×5		FANCI:NM_001113378:exon5:c.439G>T:p.G147X,FANCI:NM_018193:exon5:c.439G>T:p.G147X	CgA(+++),Syn (+++),NSE(++),inhibin (-),Vim (+),AE1/AE3 (-),CD34 (+++),Ki67 ˜ 1%	3.3484
6000000011	Female	42	Asymptomatic	Left Retroperitoneal	5.5 × 5×5	SDHB:NM_003000:exon1:c.17_18insACTCTCCTTGAGGCGCCGGTGGTCG:p.L13Wfs*58	BCL10:NM_001320715:exon2:c.136delA:p.I46fs,BCL10:NM_003921:exon2:c.136delA:p.I46fs	Vim (+),AE1/AE3 (-),CgA(+),S-100 (-),CD10 (-),CA-9 (-),TFE-3 (-),E-cadherin (-)	4.5660
6000000041	Female	59	Hyperhidrosis, hypertension	Left Adrenal Gland	5.5 × 4.7×3.5			CgA(+++),Syn (+++),Melan-A (-),inhibin (-),Vim (++),AE1/AE3 (-),CD34 (+++),Ki67 ˜ 2%	12.4804
6000000051	Male	34	Headache	Left Adrenal Gland	5 × 4.5×4	SDHB:NM_003000:exon6:c.541–2A>G		AE1/AE3 (-),Vim (+),CgA(+),Syn (+),NSE(+),S-100 (+),inhibin (+),PAX-8 (-),Melan-A (-),HMB45(-)	2.1308
6000000091	Female	53	Headache, hypertension	Left Pelvic	4.2 × 3.5×3			Vim (+),AE1/AE3 (-),CgA(+++),Syn (+),inhibin (-),Melan-A (-),CD34 (+++),Ki67<1%	14.3068
6000000151	Female	60	Palpitation, hyperhidrosis, hypertension	Left Adrenal Gland	3.8 × 3×2.5		RET:NM_020630:exon16:c.2753T>C:p.M918T,RET:NM_020975:exon16:c.2753T>C:p.M918T	CgA(+++),Syn (+++),NSE(+++),CD56 (-),inhibin (-),Melan-A (-),CD34 (+++),Ki67<1%	3.9572
60000000111	Female	51	Asymptomatic	Right Retroperitoneal	26 × 18×10		FGFR1:NM_001174066:exon11:c.1371C>G:p.N457K,FGFR1:NM_023105:exon11:c.1371C>G:p.N457K,FGFR1:NM	CgA(+++),Syn (+++),NSE(+++),CD56 (+++),Vim (+),AE1/AE3 (-),inhibin (-),Melan-A (-),CD34 (+++),Ki67(-)	1.8264

TMB, Tepresents tumor mutational burden; IHC, represents immunohistochemistry.

### Tumor lymphocyte infiltration

**FIGURE 2:**
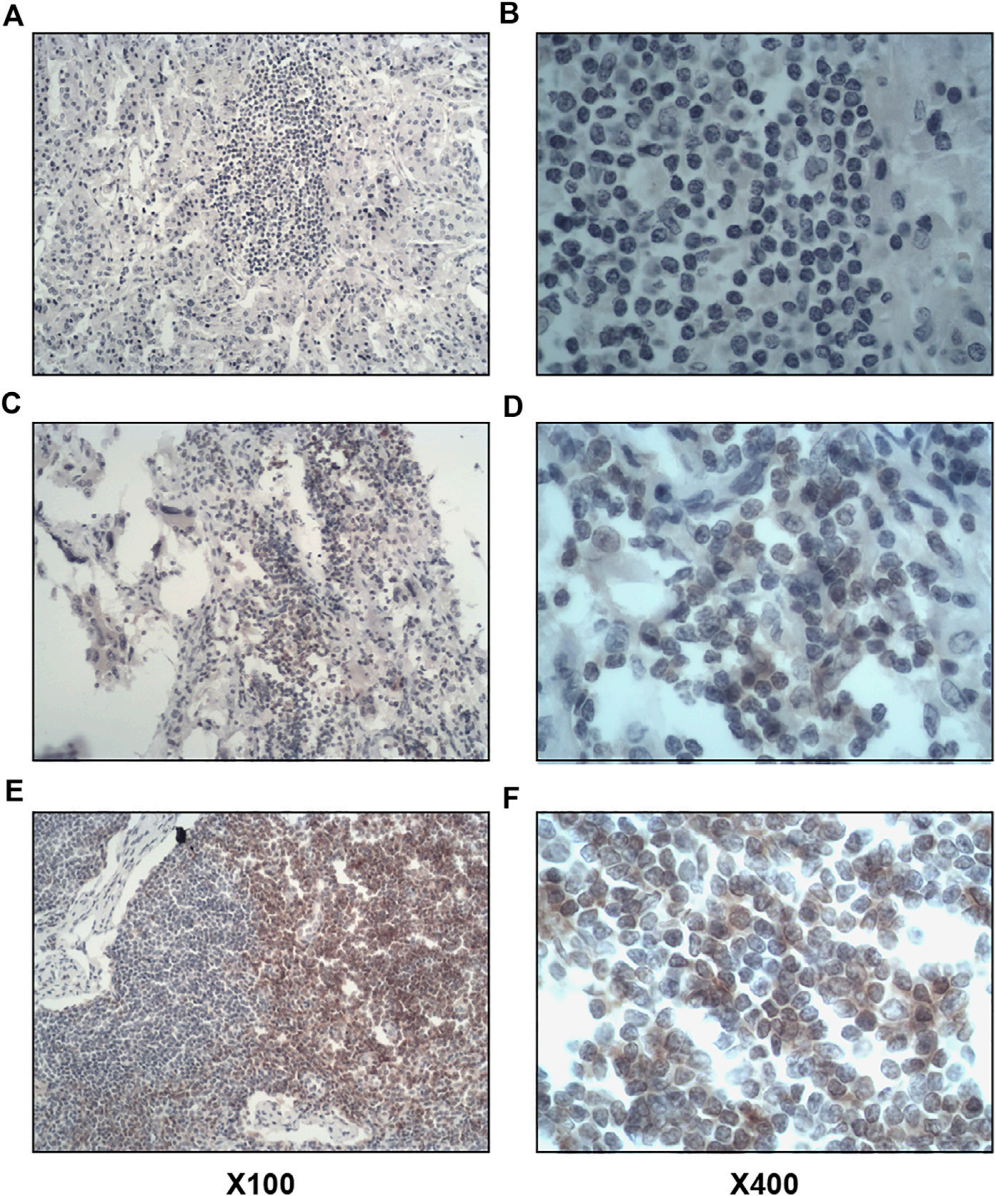
Immunohistochemical staining of CD4^+^ lymphocyte infiltration in tumor tissues. Left image × 100, right image × 400. **(A,B)**, Negative for intratumoral and stromal CD4^+^ lymphocyte infiltration. **(C,D)**, Positive for intratumoral CD4^+^ lymphocyte infiltration. **(E,F)**, positive for stromal CD4^+^ lymphocyte infiltration.

**FIGURE 3:**
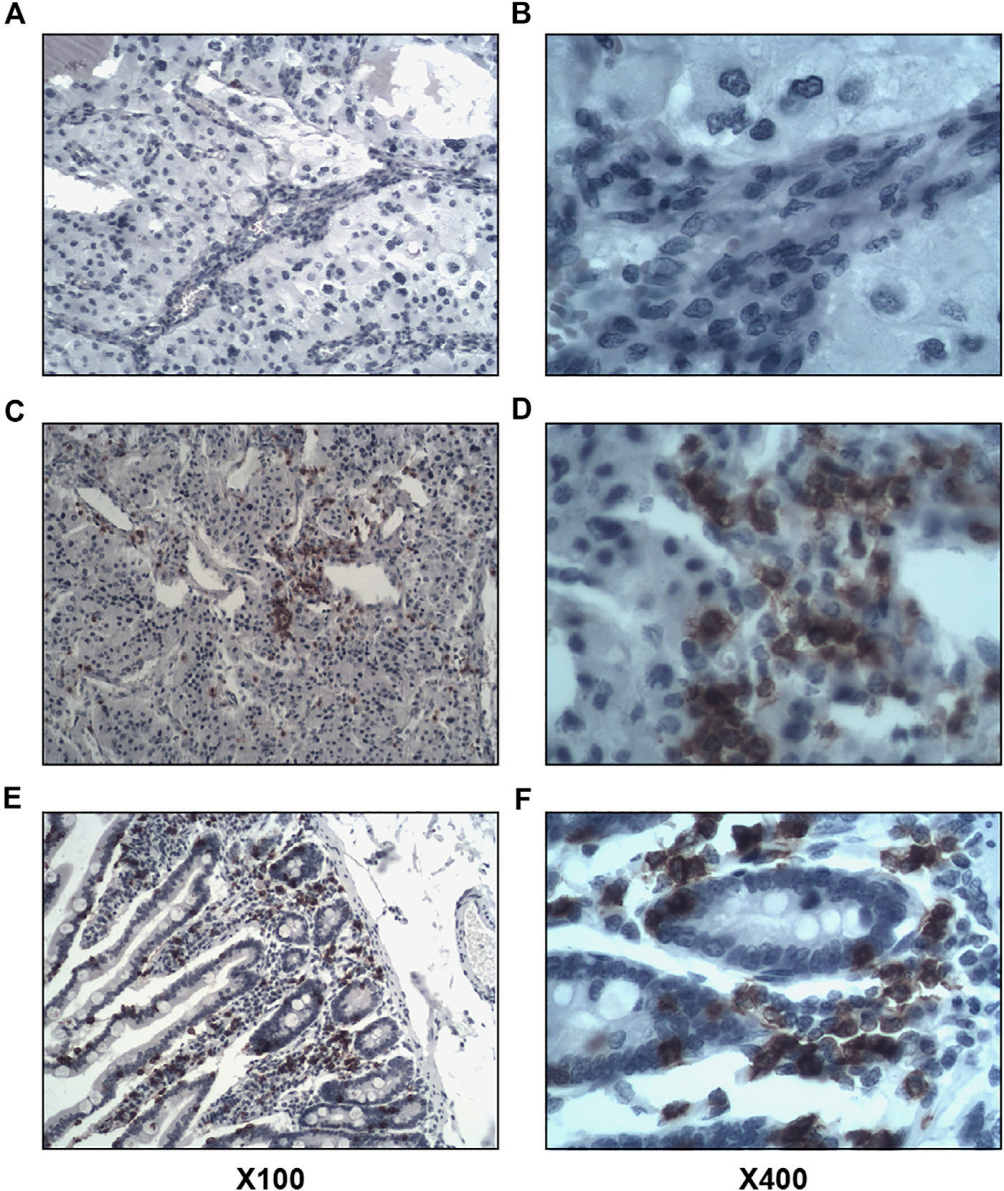
Immunohistochemical staining of CD8^+^ lymphocyte infiltration in tumor tissues. Left image × 100, right image × 400. **(A,B)**, negative for intratumoral and stromal CD8^+^ lymphocyte infiltration. **(C,D)**, positive for intratumoral CD8^+^ lymphocyte infiltration. **(E,F)**, positive for stromal CD8^+^ lymphocyte infiltration.

Of the 63 tumor tissues, 31 (49.2%) of them were positive for intratumoral lymphocyte (CD4^+^ and CD8^+^) infiltration, and 15 (23.8%) were positive for stromal lymphocyte (CD4^+^ and CD8^+^) infiltration. The results of immunohistochemical staining were shown in Figures 2, 3.

**FIGURE 4:**
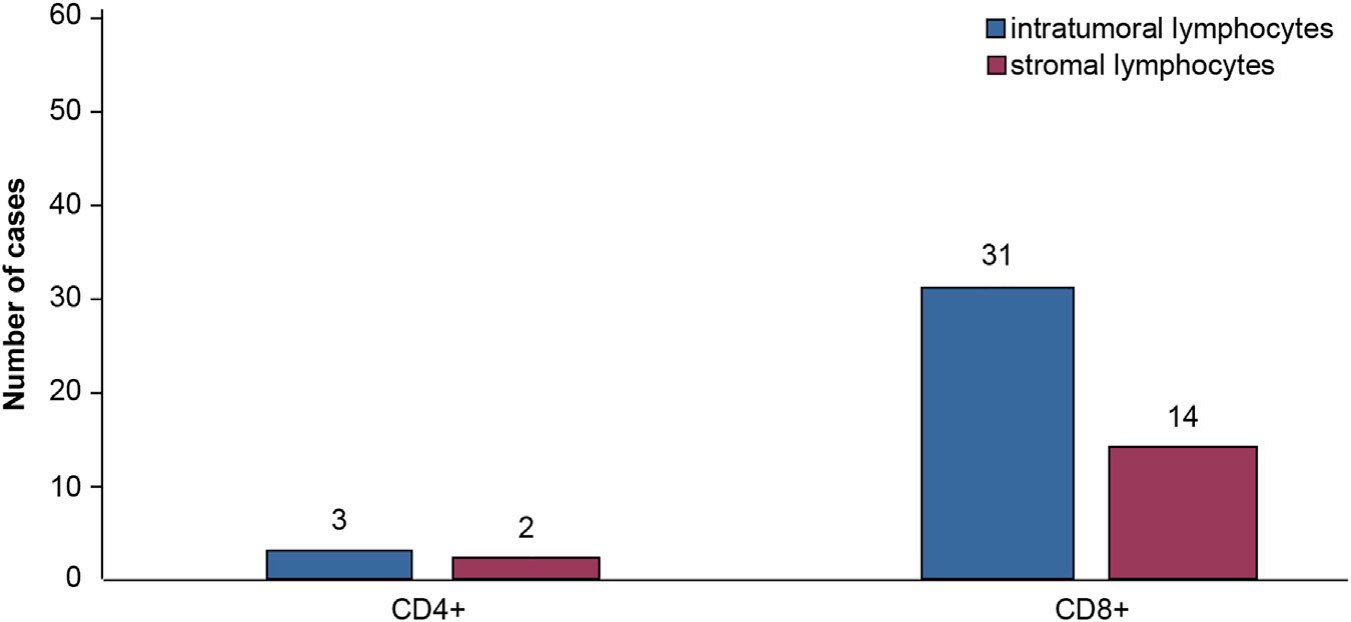
Numbers of positive cases of intratumoral and stromal CD4^+^ and CD8^+^ lymphocyte infiltration.

**FIGURE 5:**
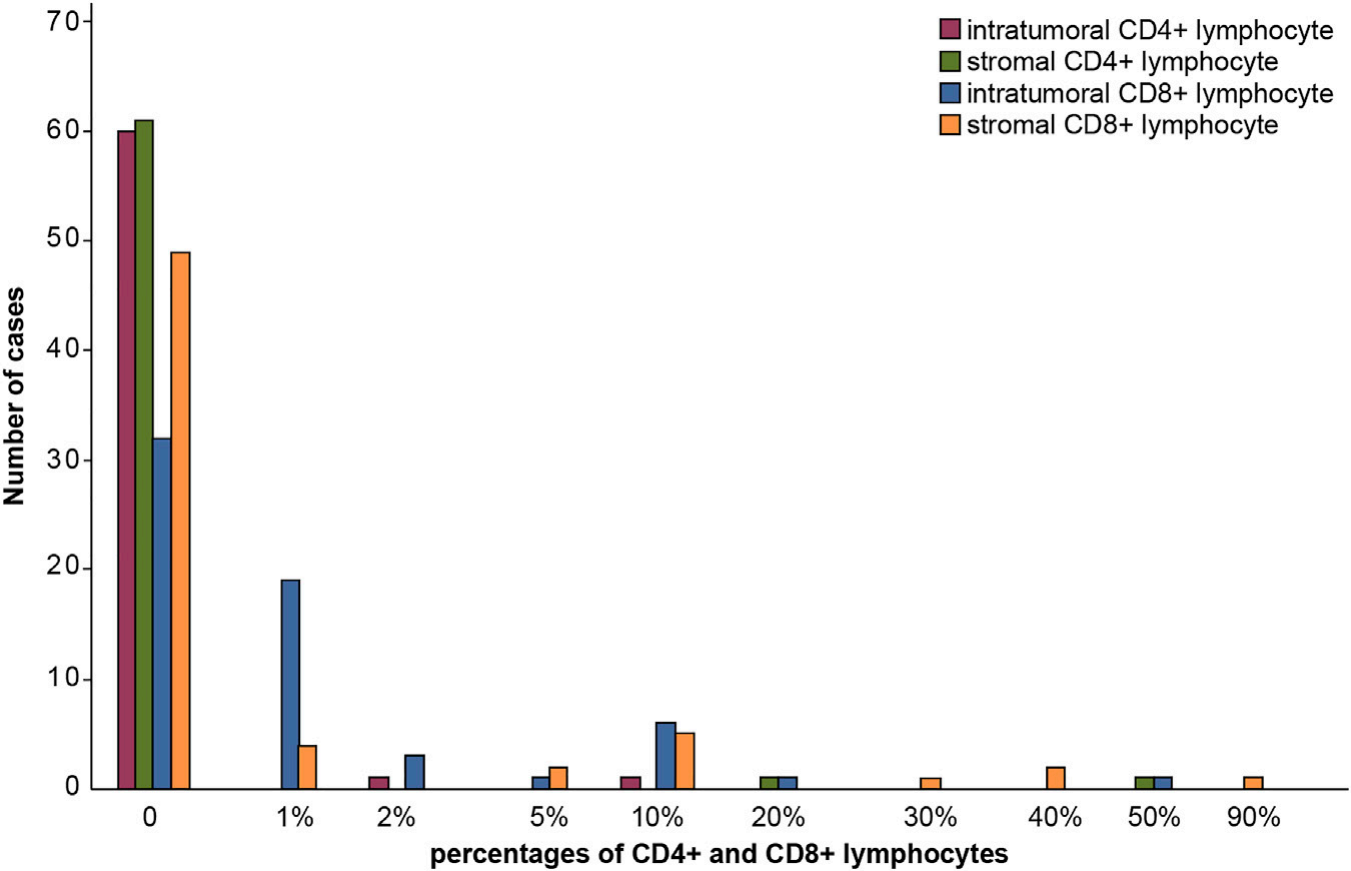
Percentage of intratumoral and stromal CD4^+^ and CD8^+^ infiltrating lymphocyte.

Moreover, we found that 3 (2.7%) cases were positive for intratumoral lymphocyte infiltration and 2 (3.1%) cases for stromal CD4^+^ lymphocyte infiltration. Additionally, we detected 31 (49.2%) cases positive for intratumoral CD8^+^ lymphocyte infiltration. Among them, 19 cases had a CD8^+^ lymphocyte percentage of 1%. Besides, we found 14 cases (22.2%) with stromal CD8^+^ lymphocyte infiltration, of which 4 cases had a CD8^+^ lymphocyte percentage of 1%. The case numbers of intratumoral and stromal CD4^+^ and CD8^+^ lymphocyte infiltration were summarized in Figure 4. The percentage of intratumoral and stromal CD4^+^ and CD8^+^ infiltrating lymphocytes was shown in Figure 5.

Intratumoral and stromal CD4^+^ and CD8^+^ lymphocytes infiltration in pheochromocytoma and paraganglioma with different degrees of malignancy.

We detected no significant differences in intratumoral CD4^+^ lymphocyte infiltration, stromal CD4^+^ lymphocyte infiltration, and intratumoral CD8^+^ lymphocyte infiltration between the metastatic, the recurrent, and the PASS-L and PASS-H groups (*p* > 0.05).

In contrast, the differences in stromal CD8^+^ lymphocyte infiltration between the metastatic, the recurrent, and the PASS-L and PASS-H groups were significantly different [9 (32.1%) *vs*. 3 (50%) *vs*. 1 (7.1%) *vs*. 0.1 (6.7%), *p* = 0.041)], as shown in Table 4.

**TABLE 4:** Case numbers and percentages of intratumoral and stromal CD4^+^ and CD8^+^ lymphocytes infiltration in. Pheochromocytoma and paraganglioma with different degrees of malignancy. Intratumoral and stromal CD4^+^ and CD8^+^ lymphocytes infiltration in metastatic and primary foci.

	Metastatic	Recurrent	PASS-L	PASS-H	*P*
Intratumoral CD4^+^	2 (7.1%)	0	1 (7.1%)	0	0.709
Tumor stromal CD4^+^	1 (3.6%)	0	1 (7.1%)	0	0.785
Intratumoral CD8^+^	15 (53.6%)	2 (33.3%)	7 (50%)	7 (46.7%)	0.886
Tumor stromal CD8^+^	9 (32.1%)	3 (50%)	1 (7.1%)	1 (6.7%)	0.041

Table 5 lists the location of the primary and metastatic foci collected from 9 cases of metastatic pheochromocytoma and paraganglioma. The differences in the positive rates and lymphocyte percentages of intratumoral CD4^+^ lymphocyte infiltration, stromal CD4^+^ lymphocyte infiltration, intratumoral CD8^+^ lymphocyte infiltration, and stromal CD8^+^ lymphocyte infiltration between the primary and metastatic foci in the metastatic group were not statistically significant (*p* ≥ 0.05), as shown in Table 6.

**TABLE 5:** Location of metastatic and primary foci.

No.	Primary foci	No.	Metastatic foci
3	Retroperitoneal	2	Mesenteric nodules
4	Bladder	5	Presacral, left iliac perivascular and right iliac perivascular fused lymph nodes
7	Retroperitoneal	6	Liver metastases and retrogastric nodules
9	Retroperitoneal	10	Pancreatic-duodenal area
13	Bladder	12	Left external iliac and right pelvic lymph nodes
15	Adrenal glands	16	Renal hilum and para-aortic lymph nodes
35	Adrenal glands	21	Renal hilum
30	Adrenal glands	30	Retroperitoneal lymph nodes
29	Retroperitoneal	33	Liver nodules

**TABLE 6:** Differential expression of intratumoral and stromal CD4^+^ and CD8^+^ lymphocytes in the primary and metastatic foci of the metastatic group.

	Primary foci	Metastatic foci	*P*
Intratumoral CD4^+^ lymphocytes	1 (11.1%)	1 (11.1%)	1.000
Stromal CD4^+^ lymphocytes	0	1 (11.1%)	1.000
Intratumor CD8^+^ lymphocytes	5 (55.6%)	6 (66.7%)	1.000
Stromal CD8^+^ lymphocytes	2 (22.2%)	6 (66.7%)	0.153
Percentage of intratumoral CD4^+^ lymphocytes	0	0	1.000
Percentage of stromal CD4^+^ lymphocytes	0	0	0.730
Percentage of intratumoral CD8^+^ lymphocytes	1% (0%–1.5%)	1% (0%–10%)	0.489
Percentage of stromal CD8^+^ lymphocytes	0% (0%–0.5%)	10% (0%–35%)	0.050

The values in the brackets represents the percentage of positive cases in the respective categories (the first four) or the range of percentage of positive cases (the last four).

### Determination of MSI status in pheochromocytoma and paraganglioma by fluorescent PCR-capillary electrophoresis

**FIGURE 6:**
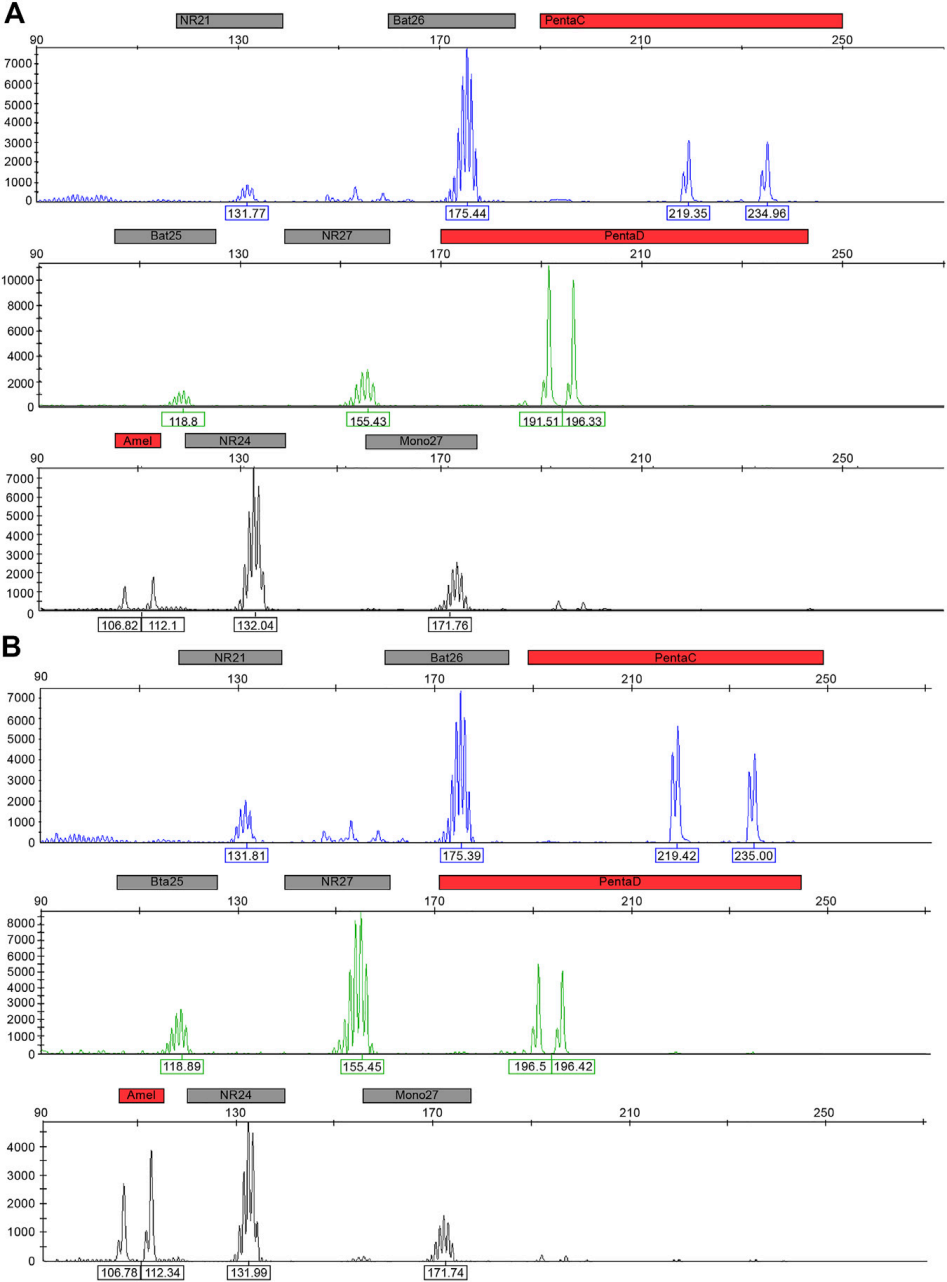
Fluorescent PCR-capillary electrophoresis detecting MSI and STR locus map typing. **(A)**. Map typing of pheochromocytoma and paraganglioma. **(B)**. Map typing of normal tissues.

We collected blood and freshly frozen tumor tissue specimens from 18 patients with PPGL and performed fluorescent PCR-capillary electrophoresis to detect MSI status. The results showed that all patients were in microsatellite stability (MSS) status (Figure 6).

### IHC detecting dMMR/MSI status of pheochromocytoma and paraganglioma

**FIGURE 7:**
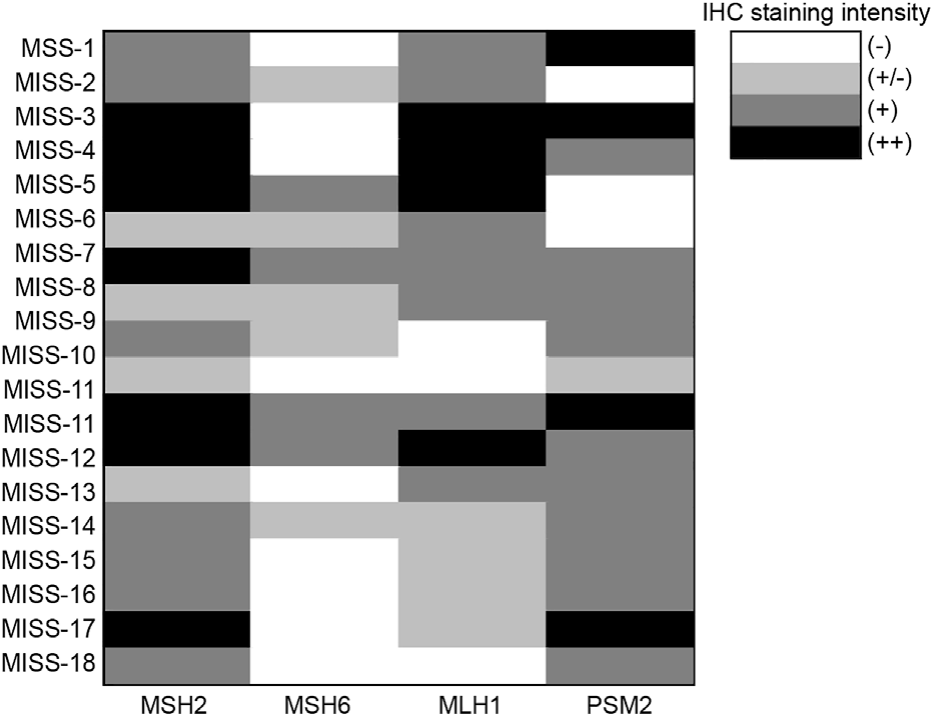
IHC staining intensity of MMR proteins in 18 cases of pheochromocytomas with MSS.

To determine the dMMR/MSI status, we stained the sections of the 18 cases of PPGL with the antibodies against MMR (MLH1, MSH2, MSH6, and PMS2). We observed that 13 cases (72%) showed dMMR (negative IHC staining for at least one MMR protein) and were of MSI-H type (Figure 7). And 5 cases (28%) showed proficient mismatch repair (pMMR), i.e., no MMR proteins with negative IHC staining (Figure 7).

**FIGURE 8:**
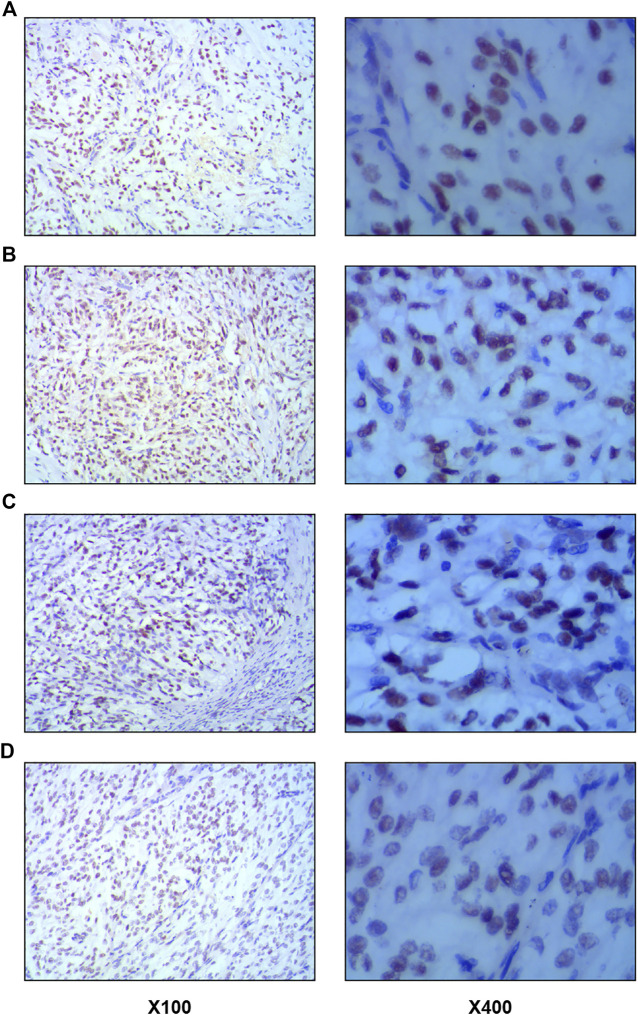
Representative images of positive immunohistochemical staining of MLH1, MSH2, MSH6 and PMS2. Left images × 100; right images × 400. **(A)**. MLH1 protein expression. **(B)**. MSH2 protein expression. **(C)**. MLH6 protein expression. **(D)**. PMS2 protein expression.

Of these 18 cases, seven only carried MSH6 deletion, three were only defective in PSM2, one only contained MLH1 deletion, and two were defective in both MSH6 and MLH1 (Figure 7). Figure 8 shows the representative images of the positive immunohistochemical staining of MLH1, MSH2, MSH6, and PMS2.

## Discussion

The 2017 WHO pathological classification classifies PPGL into malignant tumors. The new classification drops the use of “benign or malignant PPGL” and recommends adopting the “primary and metastatic malignancies” classification (Lam, 2017). The traditional definition of benign and malignant classification did not accurately reflect the biological specificity of PPGL, as a long-term follow-up survey reveals that the benign pheochromocytoma is still able to metastasize (Fishbein, 2016). To better assess the metastatic potential of PPGL, the classical PASS scoring system was used in this study, because it is the most widely used and most convenient method to categorize patients in clinical pathology, and the 12 included pathological features can reliably predict the clinical aggression of PPGL (Wu et al., 2009). Based on the scores, 29 cases of PPGL without genetic disorders and related family history of PPGL were divided into the low metastatic potential group (PASS-L) and the high metastatic potential group (PASS-H). Compared to previous studies that mostly used the traditional “benign and malignant classification”, this study adopted the new version of the pathological classification and divided the PPGL cases into PASS-L, PASS-H, recurrence, and metastasis groups to more accurately compare the differences in the immune microenvironment in PPGL with different degrees of malignancy.

In this study, immunohistochemical staining of PD-L1 performed on 63 PPGL tumor tissues showed that the overall positive rate of PD-L1 expression was only 7.9%. In previous studies, controversy arose regarding PD-L1 expression in PPGL. Pinato et al., 2017 and GUO et al., 2019 performed immunohistochemical staining on 100 and 77 PPGL tissues, revealing the positive rates of PD-L1 expression being 18.0% and 58.7%, respectively. To explain such a disparity, we reviewed the immunohistochemical results reported by the two studies. We found that based on the stringent definition of PD-L1 expression (only the localization on the tumor cell membrane is considered PD-L1 positive), some positive cases with weak and moderate PD-L1 staining reported by the above two studies should be reconsidered PD-L1 negative. Hence, the previously-reported positive rate of PD-L1 expression may be overstated. In addition, the above two studies neither performed a comparison of stratified malignancy, nor did specify the proportion of patients, resulting in an inadequate description of PD-L1 expression in PPGL. In contrast, the present study showed that the TPS in all 5 cases of PPGL with positive PD-L1 expression was only 1%, and the positive expression was not associated with PASS score stratification. Among the primary and metastatic PPGL in the metastatic group, only one primary focus had TPS of 1%. Additionally, one-third of the tumor tissues in the metastatic group exhibited positive PD-L1 expression, but only 6 cases in the recurrence group. Therefore, whether PD-L1 expression is higher in recurrent PPGL needs to be verified with a bigger sample size. Nevertheless, our study suggests that the expression level of PD-L1 is low in PPGL, and PD-L1 expression is not related to either malignancy degrees or metastatic capacities.

For the first time, this study describes the intratumoral and stromal CD4^+^ and CD8^+^ lymphocyte infiltration in PPGL with different degrees of malignancy. Our study showed that intratumoral and stromal CD4^+^ lymphocyte infiltration rarely occurs, with the highest positive rate being merely 7.1%. In cancer cells, CD4^+^ lymphocyte infiltration orchestrates the immune response to cancer (Hiraoka et al., 2006). As such, the low occurrence of CD4^+^ lymphocyte infiltration in PPGL hint at a weak response of PPGL to immunotherapy. CD8^+^ lymphocytes are a classical type of activated lymphocytes in the immune microenvironment, and CD8^+^ lymphocyte infiltration status is correlated with the prognosis of tumors, such as breast cancer. Based on our observations, the numbers of intratumoral and stromal lymphocyte infiltrations in PPGL tumors appear low. Although the total case numbers of intratumoral CD8^+^ lymphocyte infiltration were 31 (49.2%), there were 19 cases with the percentage of intratumoral CD8^+^ lymphocytes being 1%, accounting for 61.3% of the total cases of intratumoral CD8^+^ lymphocyte infiltration. Interestingly, while the difference in intratumoral CD8^+^ lymphocyte infiltration of PPGL tumors with different malignancies was not statistically significant, the difference in stromal CD8^+^ lymphocyte infiltration between the metastatic, recurrent, PASS-L, and PASS-H groups (32.1% *vs*. 50% *vs*. 7.1% *vs*. 6.7%, *p* = 0.041) was significant.

In this study, we performed dMMR and MSI in 18 cases of PPGL to determine the consistency. The results showed that all18 cases of PPGL at MSI status were microsatellite stable (MSS). This result is consistent with the results of 11 cases of PCC in Namour et al. (2006) and 27 cases of PGL in (Lehtonen et al., 2007) using 5 MSI loci (Bat25, Bat26, D2S123, D5S346, and D17S250) as references. However, in this study, the dMMR test showed that 72% (13/18) of PPGL cases were MSI-H, i.e., at least one MMR protein expression was defective. Therefore, the consistency between dMMR and MSI is low in PPGL, and dMMR testing cannot replace MSI testing to determine the microsatellite instability status of PPGL.

This study provides further insight into the inconsistency between dMMR and MSI. Four MMR proteins form two categories of heterodimers, namely MLH1-PMS2 and MLH2-MSH6. MLH1 and MSH2 are obligatory chaperones of the heterodimers, and depletion of MLH1 and MSH2 expression usually disrupts the expression of PMS2 and MSH6. Other than that, MLH1 and MHS2 can form heterodimers with other MMR proteins, such as MSH3, MLH3, and PMS1. In contrast, PMS2 and MSH6 can only form heterodimers with MLH1 and MSH2, respectively. Thus, mutations of PMS2 or MSH6 may not necessarily result in the degradation of the respective chaperones in that MSH6 can be replaced by MSH3 and PMS2 can be replaced by either PMS1 or MLH3 (Zhang and Li, 2013). In this study, we found seven specimens negative for MSH6 expression and 3 cases negative for PMS2 expression. Therefore, 77% (10/13) of the dMMR may contain the substitution of MSH6 with MSH3 or the substitution of MLH3/PMS1 with PMS2, which could, at least partially, explain the contradictory results of dMMR and MSI assays.

Kupka et al. (2008) increased the MSI-L detection rate by 9% using an expanded panel of reference loci including D2S443, D21S1436, D1S104, D3S1284, D16S752, and D11S1338, suggesting that detection of Bat25, Bat26, D2S123, D5S346, D17S250, and MONO-27 loci may not fully reflect the MSI status of PPGL. Thus, it is worthwhile to expand the microsatellite reference loci to improve the detection accuracy of MSI-H in PPGL.

Fluorescent PCR-capillary electrophoresis is a gold standard to detect microsatellite instability (Zhang and Li, 2013). Using this method, we found that PPGL is largely in the microsatellite stable state (MSS), which echoes the TMB levels previously reported in PPGL (Fishbein and Wilkerson, 2018). Therefore, we propose that the MSI status of PPGL should be detected by PCR-MSI, and that immunohistochemical staining of the four MMR proteins should not be used to assess the MSI status of PPGL in clinical diagnosis and screening. That said, it could be feasible to perform immunohistochemical staining of MSH3, MLH3, and PMS1 on specimens negative for MSH2 and PMS1 to identify the proteins that perform mismatch repair in PPGL.

Due to the low PPGL incidence, the sample size used in this study is relatively small and the time of surgery is not synchronized. So, the three characteristics of the immune microenvironment should be studied simultaneously with the same bath of specimens in future work. Moreover, the metastatic cases included in the present study were limited to lymph nodes. Therefore, liver, lung, and bone metastases should be included in the following studies. Since only two metastatic cases had metachronous recurrence (metastases found during a follow-up examination after resection of the primary foci), it is difficult to extrapolate whether PPGL was temporally heterogeneous or not in the same patient. Finally, as the TMB data obtained from the 16 patients are erratic, the sample size should be expanded to more faithfully validate the low TMB levels in PPGL.

In conclusion, our observation revealed the following characteristics of the immune microenvironment in PPGL: 1) PPGL is a microsatellite stable tumor with low TMB levels, weak neoantigen production, and poor tumor antigenicity; 2) Due to the low positive rates and low levels, PD-L1 expression is not associated with the degree of tumor malignancy; 3) The number of intratumoral infiltrating CD4^+^ and CD8^+^ lymphocytes is low. These results suggest that PPGL may be in “immune desertification” or “immune rejection” states in which lymphocyte infiltration is blocked, which could dramatically decrease the efficacy of immunotherapy. This may partly explain the results of the phase II pembrolizumab (NCT02721732) trial that reported a zero objective response rate at 27 weeks in the PPGL group (Naing et al., 2020). In the future, basic research on PPGL treatment should focus more on discovering new targeting pathways, especially the ones that are closely associated with the immune microenvironment to explore combinatory therapies. On the other hand, mechanistic studies of low PD-L1 expression and identification of other types of intratumoral infiltrating lymphocytes may help improve the efficacy of immunotherapy to treat PPGL.

## Data Availability

The original contributions presented in the study are included in the article/supplementary materials, further inquiries can be directed to the corresponding author.
